# Central serous chorioretinopathy in pregnancy

**DOI:** 10.22336/rjo.2022.68

**Published:** 2022

**Authors:** Radu Ochinciuc, Mihnea Munteanu, George Baltă, Florian Baltă

**Affiliations:** * Department of Ophthalmology, “Victor Babeș” University of Medicine and Pharmacy, Timișoara, Romania; ** Department of Ophthalmology, “Carol Davila” University of Medicine and Pharmacy, Bucharest, Romania

**Keywords:** central serous chorioretinopathy, pregnancy, micropulse laser

## Abstract

**Objective:** The objective of this work was to present two unusual cases of central serous chorioretinopathy (CSC) and the chosen therapeutic method.

**Materials and methods:** In this article, two cases of CSC in pregnant patients were described.

**Results:** The first case was a 35-year-old patient in the 16th week of pregnancy and the second one was a 26-year-old patient in the 20th week of pregnancy. Due to the contraindications associated to pregnancy, the therapeutic method chosen was subthreshold micropulse laser photocoagulation. The functional and anatomical evolution was very good in both patients.

**Discussion:** In both cases, treatment of the disease was preferred to prevent important photoreceptor losses. After the treatment, very good anatomical and functional results were obtained.

**Conclusions:** The micropulse laser is an effective solution for treating CSC. It is the only safe therapeutic solution during pregnancy. CSC can be associated with pregnancy, without necessarily suggesting pre-eclampsia.

**Abbreviations:** CSC = central serous chorioretinopathy, SRF = subretinal fluid

## Introduction

Central serous chorioretinopathy (CSC) is a maculopathy characterized by serous detachment of the neurosensory retina that affects especially young men. The most common symptoms are metamorphopsia, decreased visual acuity, central scotoma, and color vision disorders. Sometimes, it can be completely asymptomatic and remit spontaneously. 

Most of the time, the disease does not require treatment, the patients being kept under observation by monitoring visual acuity and performing optical coherence tomography (OCT) of the macula. The most recent publications suggest a significant loss of photoreceptors even in patients whose visual acuity is not significantly affected after remission or treatment of the disease [**1**,**2**]. According to these results, each of the patients with CSC should be treated to prevent significant loss of cones at the level of the macula.

Among the most important therapeutic methods of CSC are: carbonic anhydrase inhibitors (topically or orally), mineralocorticoid receptor antagonist, nonsteroidal anti-inflammatory drugs (topically or orally), argon laser photocoagulation, subthreshold micropulse laser photocoagulation, and intravitreal injections with anti-VEGF agents [**3**]. The majority of these therapeutic solutions are not compatible with pregnancy, therefore, to perform laser photocoagulation we would need an fluorescein angiography, but performing it during pregnancy is controversial [**4**-**7**].

Although CSC specifically affects males, it is also seen in women, and pregnancy is one of the risk factors. According to some studies, the incidence of CSC in pregnancy is 0.008% per year [**8**]. The exact cause is not known, but it is assumed that elevated levels of cortisol are the triggering factor [**9**]. Another aspect of CSC in pregnancy is the possibility of presenting a subretinal fibrinous exudate, in addition to the subretinal fluid typical to this maculopathy. There are publications that suggest the association of CSC with pre-eclampsia in pregnant women. Moreover, a hematocrit value > 38.0% has been shown to be a risk factor for CSC in women with pre-eclampsia [**10**]. In most of the described cases, CSC has completely remitted approximately 3 months after birth.

## Materials and methods

The purpose of this paper was to present two cases of CSC in pregnant women. Both presented accusing a decrease of visual acuity and the therapeutic solution was subthreshold micropulse laser photocoagulation. In both cases, MicroPulse™ Laser (IRIDEX, Germany) was used, and the laser power was set by the titration method, in the first case the power was 320 mW and in the second one 400 mW. 

## Results

*Case 1*

The first case was of a 35-year-old woman in the 16th week of pregnancy, who complained of a sudden decrease in visual acuity in the right eye (RE) for approximately 5 days. She was not aware of any other systemic or ophthalmological pathologies. Best-corrected visual acuity (BCVA) was 0.3 (decimal scale) in the RE and 1.0 in the left one.

**Fig. 1:**
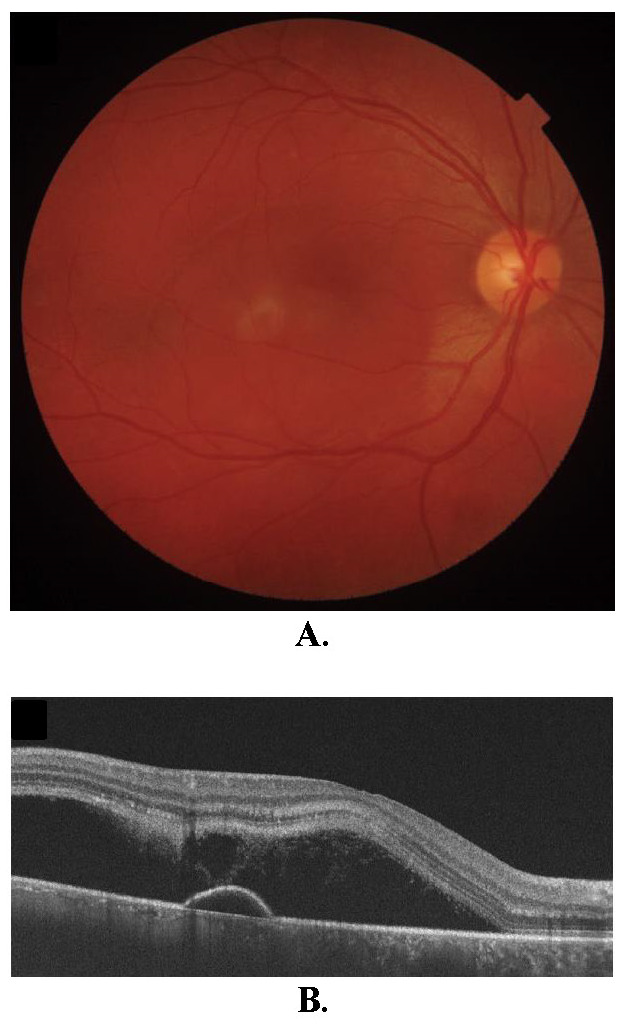
**A.** Fundus photo of the first patient. **B. ** Optical coherence tomography of the macula showing the neuroepithelium detachment and the subfoveolar hyper-reflective material

The biomicroscopic examination of the anterior pole did not reveal any pathological changes. In the posterior pole, a serous detachment of the retina and subretinal fibrinous exudates were observed in the macular area. The OCT confirmed serous detachment of the neurosensory retina in the center of the macula, as well as the presence of a subfoveolar hyper-reflective material (**Fig. 1**). Due to the low visual acuity, the increased level of subretinal fluid (SRF) and the contraindications associated with pregnancy, the chosen therapeutic solution was micropulse laser.

**Fig. 2:**
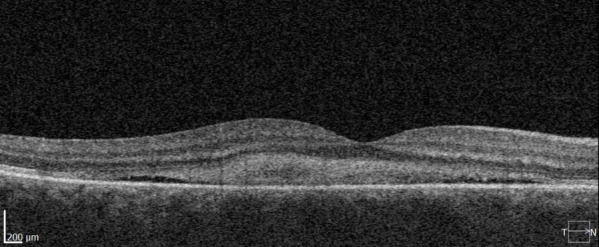
Optical coherence tomography of the first patient one month after LASER treatment

**Fig. 3:**
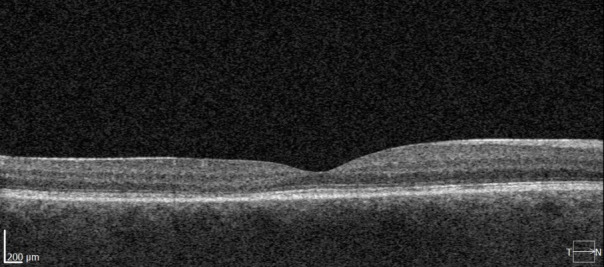
Optical coherence tomography of the first patient four months after LASER treatment

One month after the treatment, a decrease in visual acuity was observed, RE BCVA=0.05. No other changes were observed during the biomicroscopic examination. The OCT showed an important remission of the SRF and concentration of the hyper-reflective material under the fovea, which led to the decrease in visual acuity (**Fig. 2**). No other therapeutic methods were undertaken. The patient was called for a check-up after three months, when BCVA was 1.0 and the clinical aspect improved significantly. A complete resorption of the SRF and the fibrinous material was observed (**Fig. 3**). The next check-up was scheduled after birth.

*Case 2*

**Fig. 4:**
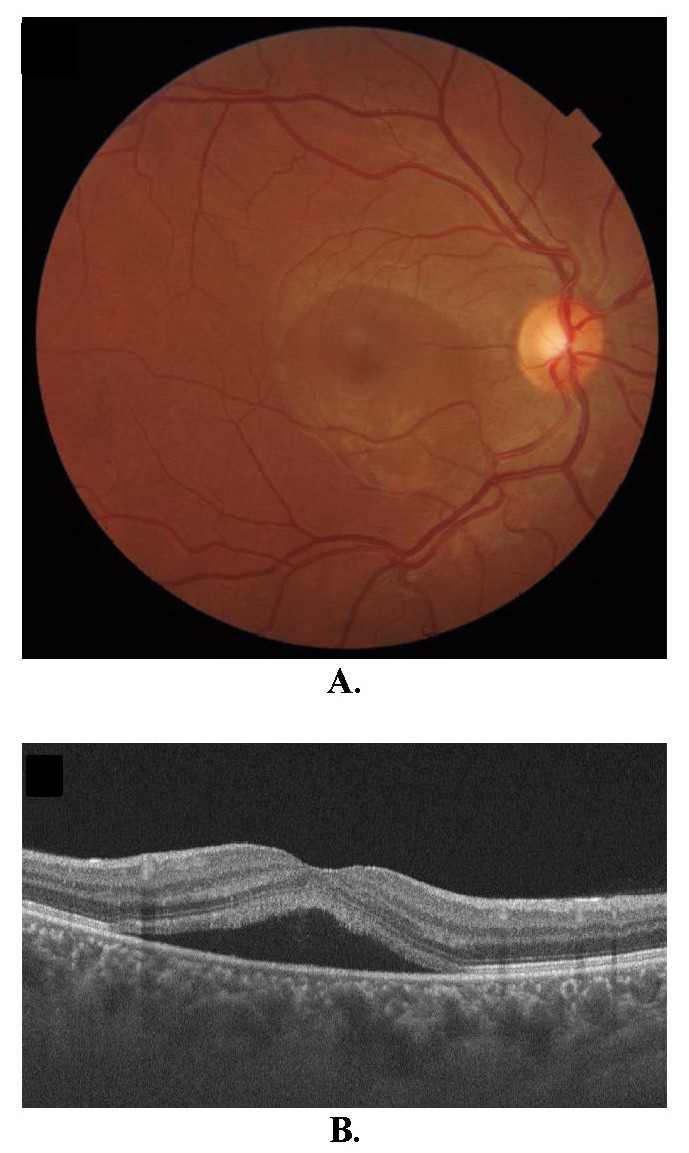
**A.** Fundus photo of the second patient. **B.** Optical coherence tomography of the macula showing the neuroepithelium detachment

A 26-year-old patient, in the 20th week of pregnancy, came in accusing a decrease of visual acuity in her RE for 3 weeks. RE BCVA=0.8 and a very good visual acuity in the LE were noted. No changes at the level of the anterior pole, and the fundoscopic examination highlighted a serous retinal detachment in the macular area. On the OCT of the macula, a significant detachment of the neuroepithelium was observed, without other changes (**Fig. 4**). The diagnosis of CSC was made and the micropulse laser was performed.

**Fig. 5:**
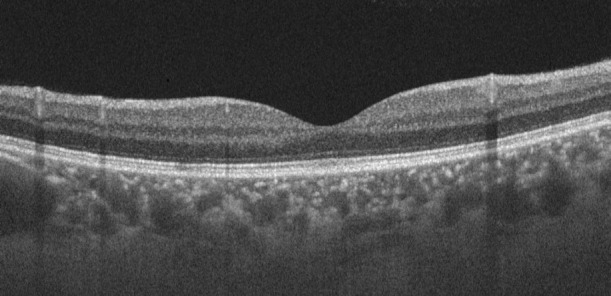
Optical coherence tomography of the second patient one month after LASER treatment

One month after applying the laser, BCVA was 1.0 and the anatomical appearance improved significantly (**Fig. 5**). The patient was called for a check-up 3 months after birth.

## Discussion

CSC is a pathology that mainly affects the males, but, to a lesser extent, women can also be affected. Pregnancy has not been shown to be an additional risk factor for CSC, the main incriminating agent being stress and the increased endogenous level of cortisol. In both cases above, the pregnancy progressed without special complications such as pre-eclampsia. Hematocrit was within normal limits in both patients.

According to the latest works published by our team, CSC causes an important loss of photoreceptors. For this reason, the current attitude is to treat all patients with this maculopathy. We preferred the therapeutic solutions that allowed us to perform the fastest resorption of the SRF, namely micropulse laser and argon laser photocoagulation. Because pregnancy is a relative contraindication for fluorescein angiography, micropulse laser remained the only effective solution for these patients. The evolution was very favorable both functionally and anatomically. Even 6 months after birth, the clinical appearance of the patients remained very favorable.

## Conclusion

The micropulse laser is an effective solution for treating CSC. It is the only safe therapeutic solution during pregnancy. CSC can be associated with pregnancy, without necessarily suggesting pre-eclampsia.

**Conflict of Interest statement**

The authors state no conflict of interest. 

**Informed Consent and Human and Animal Rights statement**

Informed consent has been obtained from all individuals included in this study. The research is in accordance with the tenets of the Helsinki Declaration, and has been approved by the review board of “Carol Davila” University of Medicine and Pharmacy, Bucharest, Romania.

**Acknowledgements**

None. 

**Sources of Funding**

The authors have no relevant financial or non-financial interests to disclose. 

**Disclosures**

None.
